# Melanin-Based Functional Materials

**DOI:** 10.3390/ijms19010228

**Published:** 2018-01-12

**Authors:** Marco d’Ischia

**Affiliations:** Department of Chemical Sciences, University of Naples “Federico II”, Via Cintia 4, I-80126 Naples, Italy; dischia@unina.it; Tel.: +39-081-674-132

## Abstract

Melanin biopolymers are currently the focus of growing interest for a broad range of applications at the cutting edge of biomedical research and technology. This Special Issue presents a collection of papers dealing with melanin-type materials, e.g., polydopamine, for classic and innovative applications, offering a stimulating perspective of current trends in the field. Besides basic scientists, the Special Issue is directed to researchers from industries and companies that are willing to invest in melanin research for innovative and inspiring solutions.

## 1. Introduction

The term melanind has traditionally been used to refer to a broad class of dark-to-black insoluble organic pigments produced by oxidative polymerization of phenolic precursors and found dwidespread in nature, from man and animals to plants, fungi and other microorganisms [1]. Traditionally investigated for their central involvement in human pigmentation and its disorders, such as albinism, vitiligo and melanoma, melanins have recently burst onto the scene of materials science, nanotechnology and biomedicine because of their unique potential for a broad range of applications [2]. What makes melanins so special and so attractive? The list of arguments is a long one. From the viewpoint of physicochemical properties, melanins share: (a) a broadband absorption spanning the entire spectral range of the visible domain; (b) a permanent electron paramagnetic resonance (EPR)d signals denoting a stable paramagnetic character; (c) hydration-dependent semiconductor-like behavior; (d) efficient dissipation of electromagnetic energy as heat. On the chemical side, most melanins display: (a) antioxidant properties, both as H-atom donor and as reducing agent; (b) metal chelation and binding of organic compounds; (c) redox behavior. In addition, melanins are bioavailable, biocompatible and biodegradable, thus representing most promising candidates for biomedical applications.

Usually, isolation from natural sources is not a practical option for the large-scale provision of melanin, since only cephalopod ink (ca. 70% pure melanin) is in principle accessible. Biotechnological approaches using microorganisms and melanin precursors may be a feasible alternative but the easiest access at present is based on the use of synthetic melanin polymers mimicking the natural counterparts. The value of this approach is exemplified by the increasing use of polydopamine, a melanin-type synthetic polymer inspired to the amine- and catechol-rich adhesive proteins of mussel byssus foot [3], for surface functionalization and coating, nanoparticle synthesis, drug delivery, sensing and other applications [4].

In this Special Issue, a collection of reports of melanin-type materials for classic and innovative applications at the cutting edge of biomedical and technological research is presented, which provides an updated panorama of emerging trends in the field. It is expected that, besides basic scientists, researchers from industries and companies that are willing to invest in melanins for innovative and inspiring solutions will benefit from the proposed selection of papers.

## 2. Synthetic and Supramolecular Control for Property Tailoring

The latest advances in melanin research have highlighted the importance of developing a complete and well-defined picture of structure-property relationships for synthetic melanin-type polymers [5]. This is a fundamental requisite for two key goals, optimization of certain existing properties or ad hoc structural modification to develop properties toward specific applications (tailoring). However, despite decades of research, several issues are still far from being definitively settled. Main difficulties arise from: (a) the heterogeneous nature of these polymers and the variable underlying levels of complexity; (b) the complex interplay of intrinsically-defined molecular properties and extrinsic aggregation-dependent features secondary to intermolecular interactions; and (c) operation of post-synthetic transformations causing more or less extensive degradation and modification of the synthetically-determined structural properties. The main consequence is that rational strategies toward property optimization and tailoring are lacking. In this Special Issue, Iacomino et al., reported the sulfur analogs of two key melanin precursors, dopamine and 5,6-dihydroxyindole (DHI) [6]. By comparing the oxidation behavior of the sulfur derivatives with that of the parent nitrogenous compounds, evidence was obtained that nitrogen is a primary determinant of structural heterogeneity in DHI-based polymers, and that replacement by sulfur is a viable entry to regioregular eumelanin-type materials for potential applications for surface functionalization by dip coating. The effect of calcium ions on the rate of polydopamine film formation and iron chelating properties of the final material was presented by Klosterman & Bettinger who emphasized the importance of post-processing conditions and salt-depending effects on the overall performance of polydopamine-based devices [7]. Büngeler et al., reviewed current knowledge on the supramolecular buildup of eumelanin and its role on the structure and controllability of materials properties, a most important issue for applications [8]. A novel method for gaining access to different particle morphologies was also presented.

## 3. Hybrid Design for Boosting Performance

Future advancements in the development of melanin-based functional materials are expected to greatly benefit from the rational design of hybrid organic-inorganic systems and from a thorough understanding of the fundamental interaction mechanisms at the interface. In this Special Issue, Pinna et al., reported a combined experimental and computational investigation aimed at modeling the melanin-porous silicon interface and at elucidating the factors controlling its stability [9]. The results showed that oxidation of the inorganic surface and the oligomer components improves adhesion and enhances photocurrent stability. A simple and green methodology for the preparation of silver nanoparticles from dopa was disclosed by Cheon & Park, who demonstrated the potential of these nanoparticles for selective detection of Pb^2+^ and Cu^2+^ in water samples [10].

## 4. Harnessing Melanin Properties for Biomedical Applications

Bioavailability and biocompatibility along with peculiar optoelectronic properties, metal chelation, drug binding and adhesion are among the key properties that spurred use of melanins for biomedical and technological applications, from bioelectronics to multifunctional devices, tissue engineering and nanotechnology for combined diagnosis and therapy (theranostics).

In this Special Issue, Solano offered a balanced overview of biomedical and biotechnological applications of melanin-type polymers with special reference to the mussel-inspired polydopamine polymer [11]. From a concise update of structural issues to biomedical applications, this review is likely to inspire new applications of melanin type materials in magnetic resonance imaging (MRI), antioxidant therapy, photothermal therapy and tissue scaffolds and sealing materials. On a more focused perspective, Longo et al., provide a stimulating review on melanin-based contrast agents for optoacoustic imaging and theranostics [12]. In particular, the authors report on optoacoustic probes that have been specifically designed to gain information on tumor vasculature, receptor expression, and tumor microenvironment. Multimodality imaging, drug delivery and therapy are among the possible applications of these melanin-based optoacoustic probes.

## 5. Conclusions: Gaps, Issues and Emerging Directions

This special issue has delineated the status quo of the research on melanin-based functional materials with special focus on the state-of-the-art and emerging directions. Some important questions would arise from a careful reading of these papers, which deserve special attention in the coming future, e.g.,: Is the development of melanin-based technology a realistic option? What hampers progress toward full maturation of this research field? What are the most promising directions?

No doubt, bioinspiration furnishes the primary conceptual framework for applied melanin research and consistently holds the key for future developments. Polydopamine is perhaps the most outstanding and paradigmatic example of how a clever use of lessons from nature may open new vistas in materials science. Today, the development of a solid melanin-based technology is a realistic opportunity but requires that some issues posed by misconceptions and false beliefs about the structure of melanins and related materials are addressed and definitively settled. The thoughtful design and/or modification of melanin precursors may be important both to probe structural issues and to invent new functional melanin-based systems on a rational basis. The enhancement of the absorption properties in the visible domain through rational control of the building block chromophores, aggregation mechanisms and overall supramolecular architecture is one important goal for some most promising applications, e.g., in optoacoustic imaging and photothermal therapy. Likewise, an in-depth understanding of the fundamental phenomena occurring at the elusive inorganic-organic interface is pivotal for the design of hybrid melanin-based systems with superior performances for, e.g., optoelectronic and bioelectronics. Additional information on melanin biopolymers and related systems can be found in another Special Issue of the International Journal of Molecular Sciences devoted to “Melanins and Melanogenesis: from Nature to Applications” edited by A. Napolitano (http://www.mdpi.com/journal/ijms/special_issues/melanins) and in a special issue of Biomimetics on “Bioinspired Catechol-based Systems: Chemistry and Applications” edited by M. d’Ischia and D. Ruiz-Molina (http://www.mdpi.com/journal/ biomimetics/special_issues/catechol).
